# Development of Microsatellite Markers Using Pyrosequencing in *Galium trifidum* (Rubiaceae), a Rare Species in Central Europe

**DOI:** 10.3390/ijms13089893

**Published:** 2012-08-08

**Authors:** Monika Szczecińska, Mirosław Kwaśniewski, Jakub Sawicki, Karolina Chwiałkowska, Kamil Szandar, Włodzimierz Pisarek

**Affiliations:** 1 Department of Botany and Nature Protection, University of Warmia and Mazury in Olsztyn, Plac Łódzki 1, Olsztyn 10-727, Poland; E-Mail: mailto:jakub.sawicki@uwm.edu.pl; 2 Department of Genetics, University of Silesia, Jagiellońska 28, Katowice 40-032, Poland; E-Mails: kwasniew@us.edu.pl (M.K.); karolina.chwialkowska@us.edu.pl (K.C.); 3 Department of Genetics, University of Warmia and Mazury in Olsztyn, Oczapowskiego 1A, Olsztyn 10-719, Poland; E-Mail: kamil.szandar@uwm.edu.pl; 4 Regional Directorate of Environmental Protection in Olsztyn, Dworcowa 60, Olsztyn 10-437, Poland; E-Mail: wlodzimierz.pisarek.olsztyn@rdos.gov.pl

**Keywords:** SSR markers, *Galium trifidum*, 454 pyrosequencing

## Abstract

We identify a large number of microsatellites from *Galium trfidum*, a plant species considered rare and endangered in Central and Western Europe. Using a combination of a total enriched genomic library and small-scale 454 pyrosequencing, we determined 9755 contigs with a length of 100 to 6192 bp. Within this dataset, we identified 153 SSR motifs in 144 contigs. Here, we tested 14 microsatellite loci in 2 populations of *G. trifidum*. The number of alleles and expected heterozygosity were 1–8 (mean 3.2) and 0.00–0.876 (0.549 on average), respectively. The markers described in this study will be useful for evaluating genetic diversity within and between populations, and gene flow between *G. trifidum* populations. These markers could also be applied to investigate the biological aspects of *G. trifidum*, such as the population dynamics and clonal structure, and to develop effective conservation programs for the Central European populations of this species.

## 1. Introduction

*Galium trifidum* (Rubiaceae) is a long-lived, perennial plant species that can be further divided into four geographically distant subspecies. Three subspecies, *G. trifidum* ssp. *columbianum* (Rydb) Hult, *G. trifidum* ssp. *subbiflorum* (Wieg.) Puff and *G. trifidum* ssp. *halophilum* (Fern. et Wieg.) Puff, grow in North America and in the Pacific islands. The fourth typical subspecies, *G. trifidum*, can be found in the boreal zone of Europe, Asia and America, where both climate and habitat conditions are optimal for its growth [1,2]. In Central and Western Europe, *G. trifidum* is a rare and endangered species. A few scattered localities of *G. trifidum* have been reported from the Polish Lowland (four localities in the Mazury Region), high Austrian mountains—1700 m a.s.l. (four localities in the Styrian Alps), France (one locality in the Pyrenees) and Turkey (one locality in the Pontic Mountains) [3–6].

In Northern Europe, *G. trifidum* occurs along ecotones of different types of water bodies (lakes, rivers, streams) characterized by changeable physical and chemical properties of water. *G. trifidum* grows there in a wide range of habitats: abundant and highly dynamic populations can be found in those providing the best growth conditions, whereas the populations reported from less optimal habitats are smaller and less vigorous. In Central Europe, *G. trifidum* prefers eutrophic moist lowland bogs and the shores of high-mountain lakes [3,4]. Due to their ecotone character, the habitats of *G. trifidum* are affected by water level fluctuations which often exert an adverse effect on the species, leading to a decline in population size or to the disappearance of entire populations.

However, studies have been conducted to investigate the ecology and population structure of *G. trifidum*. Long-term protection and management plans aiming at preserving *G. trifidum* populations should involve habitat and environmental monitoring as well as quantification of genetic diversity within and among populations. Microsatellite markers, also called simple sequence reprat (SSR) markers, are widely used in ecological studies and can also be used for investigating the genetic diversity of populations. The popularity of those markers stems from their near ubiquity, a high level of polymorphism, codominance and multi-allelic variation [7]. SSRmarkers can also be used for investigating the genetic diversity of *Galium trifidum*. In this study, we developed nuclear microsatellite markers for *G. trifidum* by using GS Junior next generation sequencing (Roche 454 Life Sciences, Branford, CT, USA). In comparison with the conventional method Sanger sequencing, pyrosequencing supports the acquisition of larger amounts of data within a shorter time.

High-throughput next generation sequencing enables the identification of even several hundred polymorphic loci in a single run [8]. To date, this technique has been successfully applied in animal, plant and bryophyte researches [9–15]. Fourteen microsatellite markers developed for *G. trifidum* are characterized in this paper. The polymorphism of SSR markers was tested in two *G. trifidum* populations of 19 and 20 individuals, respectively.

## 2. Results and Discussion

We tested PCR amplification and the level of polymorphism of the designed SSR motifs. The sequences of the SSR fragments were deposited in the GenBank (accession numbers from JX273032 to JX273045).

A single run of *Galium trifidum* DNA library sequencing in the GS Junior pyrosequencing system resulted in 144,275 reads with an average read length of 426 bp. Sequence assembling and mapping to the chloroplast genome of *Coffea arabica* allowed the alignment of 1708 reads and their contigs to the reference genome. The *Galium trifidum* sequences obtained in the analysis covered the chloroplast genome of *C. arabica* in approximately 72%, at an average depth of 2.8. The remaining reads were *de novo* assembled into 9755 contigs with a length of 100 to 6192 bp.

Analysis of the obtained sequences with the msatcommandersoftware identified 153 SSR motifs in 144 contigs. Di- (107) and tri-nucleotide (35) repeats dominated among the discovered microsatellite motifs. Longer repeat motifs included seven tetra-, three penta- and two hexa-nucleotide motifs.

Among identified SSR motifs we designed primers for 60 of them. The 14 microsatellite loci identified in the study showed a clear, single peak for each allele These 14 loci were subsequently used to screen 39 individuals collected from two populations of *G. trifidum*, one from northern Finland and one from Austria. In the studied populations, 12 loci showed polymorphism, while two loci (*Gal10*, *Gal 12*) were monomorphic (Table 1).

The number of alleles per locus ranged from one to eight, with an average of 2.3 in the Austrian population and 2.6 in the Finnish population. The expected (*H*_E_) heterozygosities ranged from 0.000 to 0.876 (0.549 on average) (Table 2). The values of coefficient H_E_ were similar in the Austrian and Finnish populations, reaching *H*_E_ = 0.438 and *H*_E_ = 0.431 respectively. Significant deviations (*p* < 0.05) from Hardy-Weinberg equilibrium (HWE) were detected for locus *Gal02* and *Gal07* in the Finnish population, which suggests the presence of null alleles. Significant LD were noted between three pairs of loci: *Gal02*/*Gal13*, *Gal04*/*Gal14* and *Gal07*/*Gal11*.

## 3. Experimental Section

### 3.1. DNA Extraction

Total genomic DNA was extracted from the leaf tissue of 20 and 19 individuals from one Finnish and one Austrian population, respectively using the DNeasy^®^ Plant Mini Kit (Qiagen, Hilden, Germany). Stems were ground with silica beads in a MiniBead-Beater tissue disruptor for 50 s, and they were subsequently processed using the manufacturer’s protocols. DNA quantity was estimated with the Qubit fluorometer system (Invitrogen, Carlsbad, NM, USA) using the Quant-IT ds-DNA BR Assay Kit (Invitrogen).

### 3.2. DNA Library Preparation and Sequencing

Eight hundred nanograms of DNA was sheared by nebulization, purified with the MinElute PCR Purification Kit (Qiagen), and subsequently processed according to the GS Rapid Library Preparation Kit Method Manual (Roche/454 Life Sciences). The quality of the DNA library was assessed by gel electrophoresis in the FlashGel System (Lonza). DNA fragments were clonally amplified using the GS Junior Titanium emPCR Lib-L Kit (Roche/454 Life Sciences). Sequencing was performed using the GS Junior pyrosequencing system, according to the Sequencing Method Manual (Roche/454 Life Sciences).

Pyrosequencing data were assembled using GS Reference Mapper software (Roche/454 Life Sciences). A two- step assembly was performed. First, the obtained sequences were assembled using the chloroplast genome of *Coffea arabica* (GenBank: NC008535) to separate chloroplast reads from nuclear reads. The remaining reads were assembled using the GS Newbler *de novo* assembler (Roche/454 Life Sciences).

The obtained contigs were searched for microsatellite motifs using MSTATCOMMANDER with default settings [16]. This program was also used for primer design. To avoid designing primers for any potential SSR locus twice, the contigs containing the same motif were compared in Bioedit 7.0.5 [17].

### 3.3. Genotyping Test

PCR reactions were performed in 20 μL of a reaction mixture containing 40 ng genomic DNA, 1.0 μM of each primer, 1.5 mM MgCl_2_, 200 μM dNTP (dATP, dGTP, dCTP, dTTP), 1× PCR buffer, 1 μL BSA and 1 U Genomic RedTaq polymerase (Sigma, St. Louis, MO, USA). The temperature profile was) initial denaturation—5 min at 94 °C, followed by 35 cycles of (2) 1 min at 94 °C, 1 min at 53 °C and 1 min at 72 °C. Final elongation was performed for 7 min at 72 °C. PCR products were separated on a QIAxcel capillary electrophoresis system, using the Qiaxcel High Resolution Kit with the alignment marker 15–500 bp (Qiagen) and the DNA size marker 25–500 bp (Qiagen). Standard OM700 settings were used as the electrophoresis program [18].

Randomly selected 14 amplicons were resequenced using amplification primers. Purified PCR products were sequenced in both directions using the ABI BigDye 1.1 Terminator Cycle Kit (Applied Biosystems, Foster City, CA, USA), and were visualized using an ABI Prism 3130 Automated DNA Sequencer (Applied Biosystems).

### 3.4. Data Analysis

Genetic diversity measures were estimated using GenAIEx 6.41 software [19]. Deviations from the Hardy-Weinberg equilibrium (HWE) and linkage disequilibrium between loci were tested using FSTAT software version 2.9.3 [20]. Significance levels were adjusted using Bonferroni correction for multiple testing.

## 4. Conclusions

The 14 microsatellite loci described here will be useful for evaluating genetic diversity within and between populations, and gene flow between *G. trifidum* populations. These markers could also be applied to investigate the biological aspects of *G. trifidum*, such as the population dynamics and clonal structure, and to develop effective conservation programs for the Central European populations of this species.

## Figures and Tables

**Table 1:** Characteristics of 14 compound microsatellite loci for *Galium trifidum.*

Locus	Repeat motif	Size range (bp)	Primer sequence	Ta (°C)	No. of total alleles	GenBank Accession No.
Gal01	(GT)_10_	336–341	F-AGGAAGAATTTAATCACGCTGG R-AGCTGAACAGGTTTGCTTG	49	3	JX273032
Gal02	(GT)_8_	268–272	F-TTCGCGTTTCCTGCAATCC R-AAACGACGGACAATGGCTC	51	4	JX273033
Gal03	(AT)_7_	306–337	F-TTTGGCAGGATAAAGGGCG R-AAACGGAAGAGTATAACAAAGGG	51	2	JX273034
Gal04	(AC)_6_	280–291	F-CACGACCAGCTGCATTTCC R-AGCTTCCTAAAGAGGCCAG	51	2	JX273035
Gal05	(AT)_6_	407–414	F-ATGGCGATTAGTGGGTCGC R-ATACGAGTCCAAAGGCAGG	53	3	JX273036
Gal06	(AAG)_5_	423–427	F-AACGGGTTGGTCAATCTCC R-GCCCTGGCGTAGAGGTG	54	4	JX273037
Gal07	(AT)_6_	419–428	F-TATGGGCGGATCGGTTGAG R-ACAAGCACTTTGATGAGACCG	53	5	JX273038
Gal08	(AT)_6_	390–398	F-TTTCTTGGGCGAAACAGGG R-GGTTAATACCAAGTTCT	55	4	JX273039
Gal09	(ATC)_6_	148–151	F-GGCTACTATTCGCCGCTTC R-AGCAAGTGAAGATTGTCATCG	53	3	JX273040
Gal10	(CCT)_5_	206	F-GACTCCGCCAAATCATCCC R-CAGAGAGTGCCACATCAGC	53	1	JX273041
Gal11	(AT)_8_	240–260	F-CATCCAACGGCAGCATAGAC R-TCCGATGAAGGTGTTGCATTC	53	5	JX273042
Gal12	(AC)_5_(TC)_5_	235	F-TCTCGTTCCCGCTCTGAAG R-GGCAGACAGTTCATGCCTC	53	1	JX273043
Gal13	(AC)_7_	425–442	F-TCTGAACTACAGCATGTTGCC R-TCTGAACTACAGCATGTTGCC	53	5	JX273044
Gal14	(AAT)_9_	423–455	F-CTGTACGCTGAGTAGCACAAG R-ACACAGCTCTTATGTAAGGCAC	53	8	JX273045

**Table 2:** Results of initial Simple Sequence Repeats (SSR) polymorphism analysis in two populations *of G. trifidum*.

	Austria *N* = 19				Finland *N* = 20			
		
Locus	*N_A_*	*A_R_*	*H*_E_	*F*	*N*_A_	*A_R_*	*H*_E_	*F*
Gal01	3	1.810	0.417	1.000	3	2.374	0.689	1.000
Gal02	3	2.351	0.674	0.636	3	2.223	0.633 ^*^	0.655
Gal03	1	1.000	0.000	0.000	2	1.883	0.533	1.000
Gal04	2	1.673	0.389	1.000	2	1.624	0.333	−0.250
Gal05	2	1.405	0.222	1.000	3	1.737	0.378	1.000
Gal06	3	2.442	0.722	1.000	2	1.845	0.500	1.000
Gal07	4	2.755	0.792	0.690	3	1.568	0.289 ^*^	0.263
Gal08	4	2.307	0.611	−0.670	3	2.089	0.550	−0.695
Gal09	2	1.908	0.556	1.000	3	1.737	0.378	1.000
Gal10	1	1.000	0.000	0.000	1	1.000	0.000	0.000
Gal11	2	1.405	0.222	1.000	3	1.737	0.378	1.000
Gal12	1	1.000	0.000	0.000	1	1.000	0.000	0.000
Gal13	4	2.527	0.667	−0.543	2	1.883	0.533	1.000
Gal14	5	2.994	0.861	1.000	5	2.948	0.844	0.690
MEAN	2.3	1.898	0.438	0.740	2.6	1.636	0.431	0.633

Number of alleles (*N*_A_), allechic richness (*A_R_*), expected heterozygosites (*H*_E_) and fixation index (*F*). A significant deviation from Hardy-Weinberg equilibrium expectations is indicated by ^*^ (*p* < 0.05).
